# Whole‐cell *Escherichia coli* lactate biosensor for monitoring mammalian cell cultures during biopharmaceutical production

**DOI:** 10.1002/bit.26254

**Published:** 2017-02-23

**Authors:** Lisa Goers, Catherine Ainsworth, Cher Hui Goey, Cleo Kontoravdi, Paul S. Freemont, Karen M. Polizzi

**Affiliations:** ^1^ Department of Life SciencesImperial College LondonLondonSW7 2AZUK; ^2^ Centre for Synthetic Biology and InnovationImperial College LondonLondonUK; ^3^ Department of BioengineeringImperial College LondonLondonUK; ^4^ Department of Chemical EngineeringImperial College LondonLondonUK; ^5^ Department of MedicineImperial College LondonLondonUK

**Keywords:** synthetic biology, whole‐cell bacterial biosensor, biopharmaceutical processing, LldPRD operon, lactate / lactic acid

## Abstract

Many high‐value added recombinant proteins, such as therapeutic glycoproteins, are produced using mammalian cell cultures. In order to optimize the productivity of these cultures it is important to monitor cellular metabolism, for example the utilization of nutrients and the accumulation of metabolic waste products. One metabolic waste product of interest is lactic acid (lactate), overaccumulation of which can decrease cellular growth and protein production. Current methods for the detection of lactate are limited in terms of cost, sensitivity, and robustness. Therefore, we developed a whole‐cell *Escherichia coli* lactate biosensor based on the lldPRD operon and successfully used it to monitor lactate concentration in mammalian cell cultures. Using real samples and analytical validation we demonstrate that our biosensor can be used for absolute quantification of metabolites in complex samples with high accuracy, sensitivity, and robustness. Importantly, our whole‐cell biosensor was able to detect lactate at concentrations more than two orders of magnitude lower than the industry standard method, making it useful for monitoring lactate concentrations in early phase culture. Given the importance of lactate in a variety of both industrial and clinical contexts we anticipate that our whole‐cell biosensor can be used to address a range of interesting biological questions. It also serves as a blueprint for how to capitalize on the wealth of genetic operons for metabolite sensing available in nature for the development of other whole‐cell biosensors. Biotechnol. Bioeng. 2017;114: 1290–1300. © 2017 The Authors. Biotechnology and Bioengineering Published by Wiley Periodicals, Inc.

## Introduction

Biopharmaceuticals are protein‐based drugs, such as monoclonal antibodies and enzymes, often with sugars attached that affect their efficacy and mechanism of action. They include blockbuster drugs such as Humira®, Avastin®, and Herceptin®, with the top five selling drugs generating nearly 70 billion USD in revenue in 2013 alone. Currently, biopharmaceuticals are predominantly produced using mammalian cell cultures to ensure appropriate glycosylation, with Chinese Hamster Ovary (CHO) cells the most prevalent host for the production of glycoproteins in industry today (Kyriakopoulos and Kontoravdi, 2013; Walsh, 2014).

The measurement of key metabolites during biopharmaceutical production is essential to inform the design of medium and feeds and to optimize cell culture conditions for increased productivity. It is also routinely used to monitor and control the trajectory of cultures during production to prolong the life of cells and maximize the yield of the product (Constantinou and Polizzi, 2013; Wuest et al., 2012). One particular metabolite of interest is lactic acid (lactate), a metabolic waste product produced as a byproduct of glycolysis. High concentrations of lactate can negatively affect cell growth and productivity (Li et al., 2010; Zhou et al., 2011) and can, via acidification of the medium, cause changes in glycosylation patterns (Yoon et al., 2005).

Lactate concentrations can exhibit very complicated dynamics across different culture phases as it is both produced, and later, consumed by CHO cells (Bertels et al., 2012). Recent work has also shown that lactate metabolism can also be an indicator of bioreactor performance during an individual run, with ability to discriminate between low and high productivity runs, even as early as the inoculum train. This suggests lactate accumulation could be a target for remedial action if cells appear to be headed towards a low productivity run. Interestingly, the analysis suggests that interventions must occur before 70 h into the production run to have the most chance of being successful (Le et al., 2012).

Given the importance of lactate as a metabolite, the development of biosensors to detect lactate in samples has been an active area of research. In particular, a number of biosensors based on enzymes such as lactate dehydrogenase or lactate oxidase have been developed. The output signals from these biosensors vary, but include spectroscopic (Shah et al., 2007), fluorescent (D'Auria et al., 2000; Li et al., 2009; Trettnak and Wolfbeis, 1989), chemiluminescent (Martinez‐Olmos et al., 2009; Roda et al., 2014; Zhou et al., 2014), or amperometric detection (Haccoun et al., 2006; Hasebe et al., 2005; Male et al., 1997; Parra et al., 2006; Weber et al., 2006), with the latter as the dominant detection modality (recently reviewed in (Rathee et al., 2016)). Novel methods of immobilization to improve detection have also been explored (Haccoun et al., 2006; Hasebe et al., 2005; Pagan et al., 2015; Tu et al., 2016; Xu et al., 2005; Yang et al., 2008). Although whole‐cell biosensors for lactate have not been previously reported, San et al. (2013) developed a Förster Resonance Energy Transfer (FRET) protein‐based biosensor, which was expressed in mammalian cells in order to directly monitor changes in intracellular lactate. The disadvantage of this approach is that it requires cells to express an additional protein, which would not be desirable in a biopharmaceutical process as it may negatively impact the ability of cells to express the recombinant product.

Current standard analytical methods for measuring lactate concentrations rely largely on HPLC and electrochemical methods, which are limited in terms of cost, sensitivity, and robustness (Bracewell and Polizzi, 2014). The industry standard instrument, the Bioprofile Analyzer, quantifies lactate concentration using an amperometric immobilized enzyme biosensor (Biomedical, 2016). Even though this analysis can be automated (Derfus et al., 2010), a large sample volume is required (at least 1 mL) and the lower limit of detection is in the millimolar range, limiting its application to later stages of culture. Enzymatic lactate detection kits, based on the lactate oxidase enzyme are also commercially available. These have a higher sensitivity for detection of lactate, but are very costly, largely due to the costs associated with purification of the enzyme.

Nature offers a potential solution in the form of bacterial genetic operons, which are designed to sense the concentration of important metabolites in the environment and activate gene expression in response (Goers et al., 2013). The sensitivity of such systems is very high—often compounds are detected at micromolar or even nanomolar concentrations (Goers et al., 2013) and a wealth of such systems that can detect important metabolites for mammalian cell culture such as sugars, amino acids, and metabolic waste products have been identified (e.g., Engels et al. (2008), Pittard et al. (2005), Senior (1975)). Therefore, bacterial genetic operons represent a rich source for designing new whole‐cell biosensors for application in industrial bioprocessing. In particular, operons to utilize lactate as a carbon source have been described in *Escherichia coli* (Aguilera et al., 2008), *Cornyebacterium* (Gao et al., 2008), *Pseudomonas* (Gao et al., 2012), and *Bacillus subtilis* (Chai et al., 2009). Any of these can be used to design a whole‐cell biosensor by co‐opting the existing genetic elements to sense lactate and activate gene expression and using them to express a reporter protein in response to lactate induction.

Research in whole‐cell biosensors began in the 1970s (Ames et al., 1973), but has recently undergone a resurgence through the application of synthetic biology methodologies to rapidly and reliably engineer new sensors (van der Meer and Belkin, 2010). The simplest whole‐cell biosensors are microorganisms, either natural (e.g., *Vibrio fischeri*, which is naturally luminescent (Bulich and Isenberg, 1981)) or engineered (Belkin et al., 1996; Quillardet et al., 1982), used to identify the presence of toxic molecules via a disturbance in the expression of a reporter protein. More sophisticated whole‐cell biosensors can be designed to sense particular compounds of interest including heavy metals (Aleksic et al., 2007; Prindle et al., 2012; Wong et al., 2015), medically relevant molecules (Auslander et al., 2014), and other small molecules (Mustafi et al., 2011; Rogers et al., 2015; Tessaro et al., 2012) and activate the expression of a reporter gene in response (thereby ‘lighting up’ in the presence of the analyte). Cell arrays can be used to detect multiple compounds from the same sample or to distinguish between related compounds (Kabessa et al., 2013; Kim et al., 2013). Although they have largely been discussed in the context of sensing environmental pollutants (Aleksic et al., 2007; Belkin, 2003; Checa et al., 2012; Prindle et al., 2012), in principle, whole‐cell biosensors can be designed to detect any compound of interest. Whole‐cell biosensors are not limited to bacteria, but can include any type of cell such as mammalian (Auslander et al., 2014; Hillger et al., 2015), plant (Wong et al., 2015), algae (Podola and Melkonian, 2003), fungi (Weitz et al., 2002), etc.

Whole‐cell biosensors have several advantages over other types of sensing formats. As a living organism, they are self‐renewing and obviate the need for purification of enzymes, leading to low reagent costs. They can be grown in large quantities from a single cell if required, meaning that they are portable. Crucially, because they are based on genetic operons, the sensitivity of whole‐cell biosensors is often very high (Goers et al., 2013). However, applying whole‐cell biosensors in complex media is difficult due to interference from other compounds in the mixture and low signal‐to‐noise ratios (Courbet et al., 2015). Previous reports of whole‐cell biosensors often lack cross‐validation with existing measurement methods (Mustafi et al., 2011) or focus on the detection of compounds in “mock” samples of low complexity (Prindle et al., 2012). Moreover, to date there have been very few reports of whole‐cell biosensors to measure metabolites that are also made by the biosensor host (Bertels et al., 2012; Mustafi et al., 2011; Tessaro et al., 2012). Instead, efforts have primarily focused on the detection of exogenous compounds (e.g., heavy metals or pollutants) (Aleksic et al., 2007; Belkin, 2003; Checa et al., 2012; Prindle et al., 2012) in order to avoid crosstalk with the host organism. However, with the advent of synthetic biology tools and techniques such as rapid and cost‐effective DNA assembly and automated part characterization, it is now possible to design biosensor variants where cross‐talk with endogenous metabolites is minimized (Wang et al., 2013).

**Figure 1:**
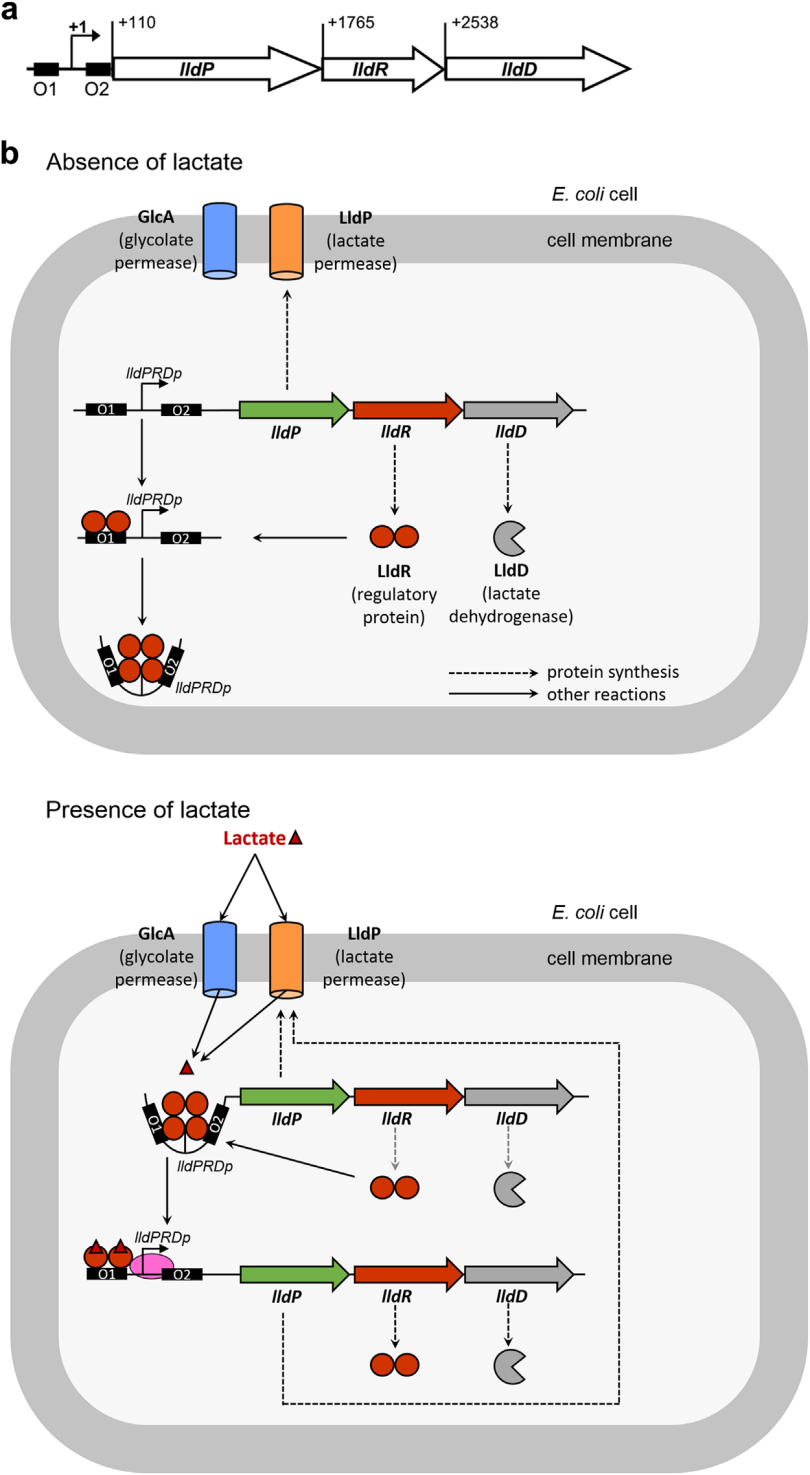
Schematic of the LldPRD operon and biochemical mechanism (**a**) Organization of the lldPRD operon. O1 and O2 represent the operator sites in the lldPRDp promoter. The three genes in the operon are (from left to right) LldP: lactate permease to allow lactate transport, LldR: regulatory protein, LldD: Lactate dehydrogenase for lactate utilization. (**b**) Diagram of the mechanism of lactate‐dependent induction of lldPRD operon in *E. coli* cells. Top: In the absence of lactate, dimers of LldR bind to the operator sites in the lldPRDp promoter and form a tetramer, sequestering the DNA and preventing transcription of the operon. Bottom: Lactate enters the cell via the glycolate permease (GlcA) or LldP and interacts with the LldR regulator protein. The LldR dimer bound to O2 dissociates when bound to lactate, but the dimer bound to O1 becomes a transcriptional activator that promotes transcription of the operon when lactate binds.

The application of whole‐cell biosensors to bioprocess monitoring would require quantification of the concentration of metabolites as obtained by the Bioprofile Analyser, HPLC, and other analytical methods. However, this type of characterization is not routinely conducted in the development of new biosensors and is complicated by the complexity of the sample background. Using iterative cycles of characterization in increasingly complex environments, we developed a workflow to achieve robust quantification of lactate in mammalian cell culture samples. Crucially, we used automation to control the environment, which will increase reproducibility and limit the effects of crosstalk with the host organism by keeping cells in the same metabolic state across different experiments. We used two genetic variations of the natural *E. coli* lldPRD lactate operon (Fig. 1) to construct our biosensor and tested its performance in a wide range of conditions. We were able to demonstrate that our whole‐cell bacterial biosensor shows a measurable response to lactate in a variety of different cell culture media. Further, we show that the concentration of lactate in real cell culture samples can be accurately quantified when validated against existing analytical methods. Finally, our biosensor has a lower limit of detection than the Bioprofile® Analyzer and is less expensive per sample analyzed than commercially available lactate oxidase assay kits. Therefore, it can be used for routine monitoring of cell culture trajectories, even in early phases of culture where lactate concentrations may be less than 1 mM.

## Materials and Methods

### Molecular Biology

*E. coli* DH5α (α‐Select, Bioline) cells were used for expression and characterization of all bacterial constructs. All plasmids were based on the pSB1A2 plasmid backbone and pre‐existing BioBrick parts were obtained from the distributions of the iGEM Registry of Standard Biological Parts (partsregistry.org). The lldPRD promoter and the lldR gene (Supplementary Table SI) were amplified from the *E. coli* genome via PCR using the primers listed in Supplementary Table SII, which incorporate the BioBrick prefix and suffix. Constructs were assembled using the BioBrick Assembly standard. Plasmids were verified by DNA sequencing (Source Bioscience, Nottingham, UK).

### Biosensor Characterization

Biosensors were characterized using an Aviso‐GmbH Theonyx robotic platform linked to a plate reader (Synergy HT, BioTek Instruments, Inc., Winooski, VT) and a shaking incubator (Ventura 2000, Mikura). Single colonies containing the biosensor plasmid or a control plasmid lacking the lldPRD promoter were used to inoculate 1 mL of LB medium containing 100 μg/mL ampicillin (Sigma–Aldrich) and grown for approximately 6 h during the day at 37°C with shaking at 250 rpm. The starter culture was diluted 1:100 into 5 mL of M9 minimal medium (1x M9 salts, 2 mM MgSO_4_, 0.1 mM CaCl_2_, 0.34 g/L thiamine hydrochloride, 1 g/L NH_4_Cl, and 0.4% glycerol or glucose as appropriate) containing 100 μg/mL ampicillin and grown overnight at 37°C with shaking. The next morning, cultures were transferred to 96‐well plate format with 200 μL per well. Using the Theonyx platform, the cells then underwent dilution into fresh M9 medium containing 100 μg/mL ampicillin at a cell density of 140 ng/μL (OD600 of 0.15 in the attached plate reader) in a total volume of 100 μL. This was followed by an outgrowth phase of approximately 2.5 h to allow cells to attain exponential growth. Subsequently, 25 μL of sample were added. For characterization, mock samples containing varying concentrations of L‐lactate, D‐lactate, or pyruvate (all Sigma–Aldrich) were mixed in appropriate background solvent of M9 minimal medium, CD‐CHO (Invitrogen, ThermoFisher Scientific, Waltham, MA), CD‐CHO + HT supplement (11067–030, Gibco) + 8 mM glutamine (ThermoFisher Scientific), DMEM (SIGMA, 51449C), DMEM containing phenol red (Sigma, D6546), or DMEM containing 10% (v/v) calf bovine serum (ATCC, 30–2030). For quantification of mammalian cell culture samples, culture supernatant taken from growing cultures described below were added and a standard curve containing known concentrations of lactate in the same background solution was included to create a transfer function from which lactate concentration was calculated. The OD_600_ and GFP fluorescence (λ_excitation_ 485/20 nm, λ_emission_ 528/20 nm) were measured every 10 min. All cell growth and characterization on the robotic platform was done at 37°C with shaking at 700 rpm.

### Analysis of Biosensor Data and Calculation of Concentration Estimates

Raw fluorescence data were processed in R 3.1.2 as follows. A spline interpolation was fit to each fluorescent time course to reduce the impact of equipment error on the values. To prevent over‐fitting of the spline and ensure that the interpolation calculated was a strong predictor of the original time course, an exhaustive cross validation was carried out to choose the ideal smoothening value for each. Leave‐p out cross validation was used, where *p* = 2. Subsequently, Microsoft Excel was used for linear regression of the fluorescence versus corrected OD_600_ of the control cells was used to fit an equation, which was used to subtract cellular autofluorescence from biosensor fluorescence measurements as follows:

$${Fl}_{i,corrected}={Fl}_{i}-((m\times {OD}_{i})+c)$$

where *m* and *c* represent the slope and intercept of the trendline, respectively.

The fluorescence rate of change (GFP synthesis rate) was calculated by dividing the difference in fluorescence between adjacent time points by one half of the difference in OD_600_ between the same time points. All samples were normalized to the biosensor cultures without addition of lactate. The transfer functions were created using data from 150 min post addition of the sample to the biosensor cultures using the GraphPad Prism nonlinear regression (sigmoidal, 4PL) function to fit the data.

To compare the biosensors under different conditions, the fold‐induction, goodness‐of‐fit of the transfer function, limit of detection, and sensitivity were calculated. All values were calculated at 150 min, except for the experiment where cells were grown directly in CD‐CHO medium, where 90 min was used due to lack of separation of the lactate concentrations at 150 min. The fold‐induction was calculated as the fluorescence rate of change of samples containing 14 mM lactate divided by the fluorescence rate of change of samples with no added lactate. The goodness‐of‐fit and the sensitivity (also called the ‘Hill slope’ or the gradient of the Hill function) were calculated using GraphPad Prism. The limit of detection reported is the lowest lactate concentration that gave a response different from background at the 150 min timepoint. The actual lowest limit of detection may be lower, but was not tested here or appears at a different time point.

To calculate lactate concentrations in mammalian cell culture samples, GFP synthesis rate values from a set of standards from the same experiment were chosen to make a standard curve and the GFP synthesis rate values from biosensors incubated with the cell culture samples were used to interpolate (sigmoidal, 4PL) the lactate concentrations using the GraphPad Prism software®.

### Mammalian Cell Culture

#### Hybridoma Flask Cultures

ATCC‐CRL1606 murine hybridoma, producing a mAb (HFN7.1) against human fibronectin were cultured in 500 mL Erlenmeyer flasks (Corning, UK) at 37°C in an atmosphere containing 5% CO_2_ on an orbital shaking platform rotating at 125 rpm. The basal growth medium was 100 mL glutamine‐free DMEM (Sigma, D6546) supplemented with 10% (v/v) calf bovine serum (ATCC, 30–2030) and 0–10 mM Gln (Sigma, G3126), which was sterilized by filtration.

#### CHO‐S Flask Cultures

The CHO‐S cell line expressing a truncated glycosyltransferase‐ECFP‐EYFP fusion protein was a kind gift from Antony Constantinou (CSynBI, Imperial College London). The CHO‐S cells were cultured in a 250 mL Erlenmeyer flask (Corning) at 37°C in an atmosphere containing 5% CO_2_ on an orbital shaking platform rotating at 125 rpm. The basal growth medium was 100 mL CD‐CHO medium (Invitrogen), supplemented with 8 mM glutamine (Invitrogen) and 1× HT supplement (11067–030, Gibco).

#### GS‐CHO Bioreactor Cultures

The GS‐CHO 46 cell line, kindly provided by Lonza Biologics (Slough, UK), was cultured at 140 rpm in humidified 37°C incubator with 8% CO_2_ supply. Cells were sub‐cultured in fresh CD‐CHO medium (Life Technologies, Paisley, UK) every 3 days at a seeding density of 2 × 10^5^ viable cells/mL and transferred into the bioreactor system on the fourth cell passage. Twenty‐five micrometer L‐Methionine sulfoximine (MSX) (Sigma–Aldrich, Dorset, UK) was added to the medium in first and second passages only.

The 3L Mobius® CellReady bioreactor (EMD Millipore, Bedford, MA) was inoculated at a seeding density of 3 × 10^5^ cells/mL in an initial culture volume of 1.2 L. The culture was mixed using an in‐house up‐pumping marine impeller rotating at 100 rpm. The culture was controlled with the my‐control unit (Applikon Biotechnology, Schiedam, the Netherlands) at a temperature of 37 ± 0.5°C using a heating blanket, and at a pH of 7.0 ± 0.1 using CO_2_ supplementation and 100 mM NaHCO_3_/100 mM Na_2_CO_3_ alkali solution (Sigma–Aldrich). Dissolved oxygen tension (DOT) was set at a minimum of 30% with oxygen supply. Culture temperature, pH, and DOT were monitored online using BioExpert software, version 1.1X (Applikon Technology, Schiedam, the Netherlands). The culture was supplemented with CD Efficient™ Feed C AGT™ (Life Technologies) at 10% of culture volume on alternate days starting from day 2. 5% w/v antifoam C (Sigma–Aldrich) was added when foaming was observed. The working volume was maintained between 1.0 and 1.3 L by drawing out excess culture fluid. The cell culture was harvested on day 16.

### Cross Validation of Lactate Concentration

Samples of 1–5 mL as required were taken from the culture, the cells were removed by centrifugation at 800 rpm for 5 min and the supernatant transferred to a fresh tube and stored at −20°C before further analysis. The Bioprofile^®^ 400 Analyzer (Nova Biomedical, Waltham, MA) was used to measure ammonia, glucose, glutamine, glutamate, and lactate concentrations according to the manufacturer's guidelines. Lactate concentrations in samples were also analyzed using a colourimetric, enzymatic lactate kit (MAK064, Sigma) according to the manufacturer's instructions.

## Results

### Initial Testing of Biosensor Designs

**Figure 2:**
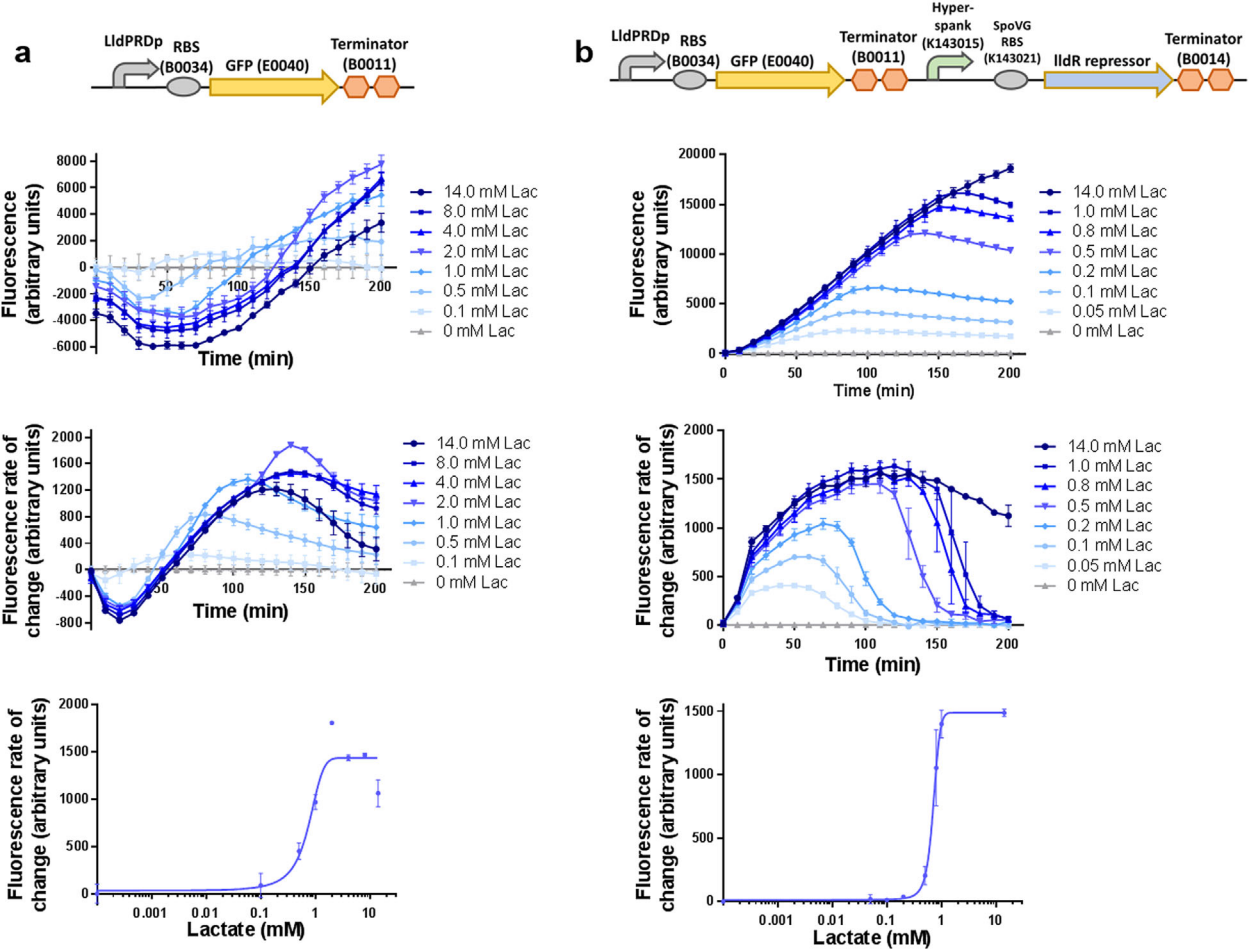
Characterization of two different biosensor designs (**a**) Biosensor without overexpression of LldR (**b**) Biosensor with constitutive overexpression of LldR regulator. In each panel: (Top): Diagram of the genetic construct. (Bottom): Characterization data at different concentrations of lactate: Fluorescence normalized by OD600, fluorescence rate of change, and transfer function from data at 150 min. Error bars represent the standard deviation of six measurements (two technical replicates of three biological replicates). The negative fluorescence and fluorescence rate of change values are a result of the normalization of data to the biosensor culture without added lactate.

We designed and constructed two whole‐cell lactate biosensors (Fig. 2) and compared their performance in samples of M9 minimal medium with glycerol as the carbon source spiked with different concentrations of L‐lactate. The first biosensor expressed green fluorescent protein (GFP) under the control of the lldPRD promoter (Aguilera et al., 2008). Characterization of this construct showed a very limited lactate response (Fig. 2a, Table I) with only twofold induction and a high level of leaky expression in the absence of lactate. For example, the fluorescence rate of change of the biosensor at 150 min is 1402.8 arbitrary units versus 25.3 arbitrary units in the improved biosensor design discussed below (data not shown), indicating that the genomically expressed lldR is not sufficient to repress reporter expression in the absence of inducer. Negative values for fluorescence and GFP synthesis rates result from the data analysis procedure where values are normalized to the biosensor cultures with no added lactate. In the case of this biosensor design, low concentrations of lactate are not distinguishable from zero lactate and, by random chance, can give fluorescence values below that of the culture without added lactate.

**Table I:** Comparison of biosensor across different growth media and sample composition

Biosensor	Growth condition	Sample background	Fold‐induction	Transfer function goodness‐of‐fit	Limit of detection	Sensitivity (hill slope)
Without llDR	M9 with glycerol	M9 with glycerol	2.02	0.905	0.5	1.54
With lldR	M9 with glycerol	M9 with glycerol	59.67	0.999	0.05	4.00
With lldR	M9 with glucose	M9 with glucose	18.63	0.981	0.05	4.62
With lldR	M9 with glycerol	D‐lactate in M9 with glycerol	1.95	0.974	0.5	0.34
With lldR	M9 with glycerol	Pyruvate in M9 with glycerol	0.73	0.837	0.5	‐11.57
With lldR	CD‐CHO	CD‐CHO*	2.78	0.971	0.2	0.45
With lldR	M9 with glycerol	CD‐CHO	8.25	0.999	0.1	0.09
With lldR	M9 with glycerol	CD‐CHO + HT supplement, 8 mM gln	11.96	0.992	0.05	0.28
With lldR	M9 with glycerol	DMEM	16.75	0.771	0.001	0.66
With lldR	M9 with glycerol	DMEM + phenol red	23.11	0.950	0.1	0.29
With lldR	M9 with glycerol	DMEM + serum	7.90	0.947	0.05	0.50

*The transfer function for this sample was calculated from the data at 90 min post addition of lactate.

An improved version of the biosensor contained an additional module overexpressing the lldR transcription factor under a constitutive promoter in order to lower the baseline GFP expression. Characterization of this biosensor in M9 minimal medium with added lactate showed a clear lactate response with good separation between different concentrations (Fig. 2b, Table I). The new biosensor had a 60‐fold induction and a lower limit of detection that was an order of magnitude lower. Thus, the version of the biosensor with the additional module for overexpression of lldR was used in all further studies.

The biosensor sensitivity to the growth environment was also tested (Supplementary Fig. S1a, Table SI). When cells were grown in M9 minimal medium with glucose instead of glycerol as the carbon source, the biosensor is still able to detect lactate with very similar lower limit of detection. However, the induction of the response is nearly 70% lower suggesting the environment does influence sensing capabilities.

We also analyzed the biosensor response to two structurally similar metabolites. The biosensor was found to show a very small response to D‐lactate (Supplementary Fig. S1b, Table SI) with a sensitivity decreased by 91% and a lower limit of detection of 0.5 mM. However, its response to pyruvate was within the inherent measurement error and therefore negligible (Supplementary Fig. S1c, Table SI). It is possible that the response to D‐lactate is due to small amounts of L‐lactate contamination in the D‐lactate, which is only 99% pure. The fact that the biosensor can distinguish between L‐lactate and pyruvate is important for cell culture studies where pyruvate is often present either as a medium additive or additional metabolite produced by the mammalian cells during metabolism.

### Developing a Workflow for Quantification

**Figure 3:**
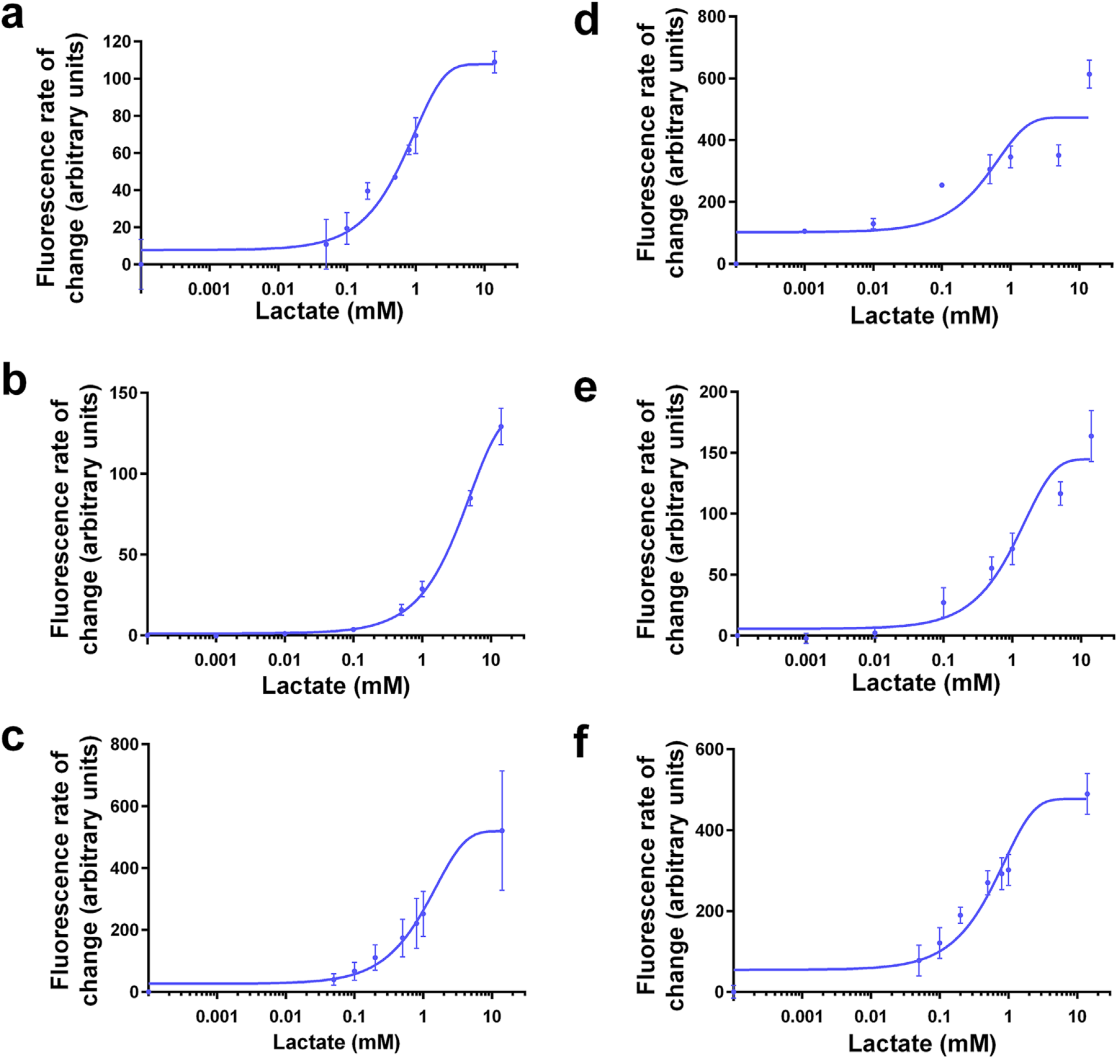
Measurement of lactate in mammalian cell culture media (**a**) Biosensor response to L‐lactate when grown in CD‐CHO medium. (b‐f) Biosensor response to L‐lactate spiked into different mammalian cell culture media (**b**) CD‐CHO medium, (**c**) CD‐CHO medium with added HT supplement and 8 mM glutamine, (**d**) DMEM medium, (**e**) DMEM medium with added phenol red, (**f**) DMEM medium with added serum. Data are presented as transfer functions from data at 150 min. For the time course data please see Supplementary Figure S2. Error bars indicate the standard deviation of six measurements (two technical replicates each of three biological replicates).

When biosensor cells were cultured directly in CD‐CHO, a widely used chemically defined mammalian cell culture medium, the lower limit of detection was decreased by a factor of 4 and the GFP synthesis profile showed only a short burst which quickly returned to baseline (Supplementary Fig. S2a), most likely due to the rapid growth rate of the cells in this medium. In contrast to the robust signals in M9 medium, the low signal‐to‐noise ratio of cells cultured in CD‐CHO (fold induction of 2.8 vs. 60, Fig. 3A, Table I) made it difficult to distinguish between low concentrations of lactate. This is analogous to the observations of Courbet et al. (2015) where signals were damped in urine and serum due to the complexity of the medium. Although CD‐CHO is chemically defined, it contains more than 70 different compounds in its formulation (European Patent Application, EP 1 482 031 A1, 2004) including sugars, amino acids, and lipids.

Therefore, we developed a workflow where the biosensor cells were initially cultured in M9 medium and a mock sample of mammalian cell culture medium supplemented with a known concentration of lactate was added to the cells. By minimizing the volume of the cell culture sample to 20% of the total volume (fivefold dilution), the biosensor cells were able to detect lactate spiked into CD‐CHO medium (Fig. 3b, Table I), CD‐CHO medium containing HT supplement and glutamine (Fig. 3c, Table I), Dulbecco's Modified Eagle Medium (DMEM) (Fig. 3d), DMEM containing the pH indicator phenol red (Fig. 3e, Table I) and DMEM containing 10% serum, a common culture additive (Fig. 3f, Table I). Although the dynamic (Supplementary Fig. S2a‐f) and static (Fig. 3a–f) response of the biosensor to lactate changes in different media, a lactate response is always measurable, and therefore, quantifiable using a standard curve.

In all conditions, the lactate response initially increases, but levels off over time. Further investigation suggests that this is as a result of lactate concentration in the biosensor cultures decreasing over time (Supplementary Fig. S3), most likely due to lactate being taken up and metabolized by the bacterial cells as part of the sensing process. The *E. coli* strain used as a biosensor host retains the genomic copy of LldP (lactate permease) and LldD (lactate dehydrogenase), which allow for the transport and utilization of lactate, respectively, by the biosensor cells when the operon expression is induced (Fig. 1).

### Utility Testing With Cell Culture Samples and Cross Validation

**Figure 4:**
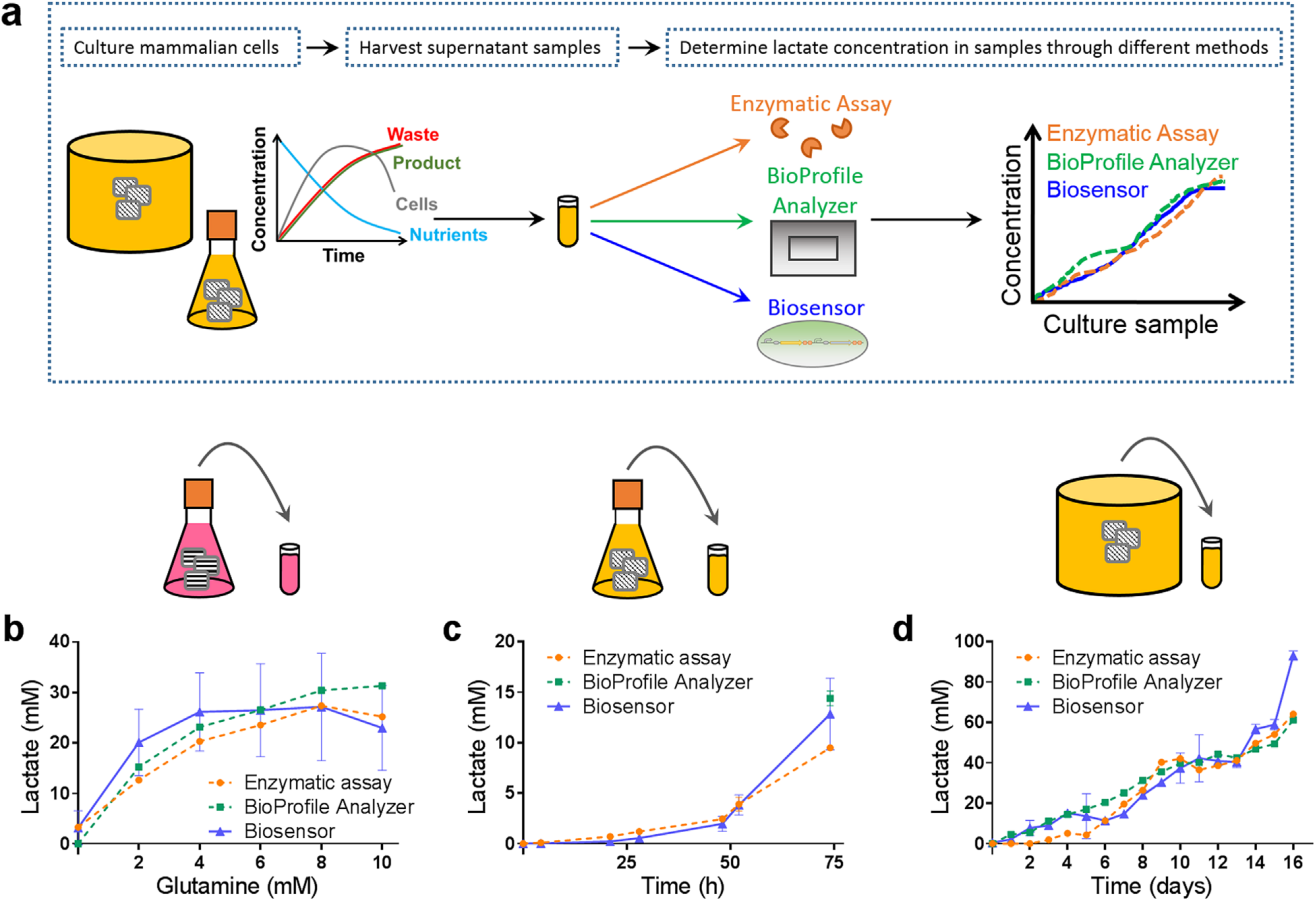
Quantitative analysis of cell culture samples using lactate biosensor with cross‐validation against existing methods. (**a**) Experimental workflow. Samples were collected from mammalian cell cultures and analyzed for lactate content using the lactate biosensor (blue), a BioProfile^®^ Analyzer (green) and a lactate oxidase enzymatic assay (orange). (**b**) Samples from CRL1606 hybridoma cultures in DMEM containing phenol red and 10% serum. Six hybridoma cultures were run in parallel with varying starting concentrations of glutamine starting as indicated in order to maximize antibody titre. The samples shown here were collected after 100 h of mammalian cell culture. (**c**) Time course samples from a CHO‐S batch shake flask culture in CD‐CHO medium supplemented with 1X HT supplement and 8 mM glutamine. (**d**) Time course samples from a GS‐CHO fed‐batch bioreactor culture in CD‐CHO supplemented with Feed C every two days. Error bars indicate the standard deviation of six measurements (two technical replicates each of three biological replicates) for biosensor measurements, two technical replicates for the enzyme assays and a single measurement for the BioProfile^®^ Analyzer. For further metabolite data for these mammalian cell cultures please see Supplementary Figures S4 and S6. Missing values are below the limit of detection of the BioProfile® Analyzer.

We then undertook a series of cell culture experiments to see if the biosensor could be used to quantify lactate metabolism in real cultures. When cells are inoculated into the medium, they use the nutrients provided, grow, produce additional metabolites, and express recombinant protein, further increasing the complexity of the sample. To test the performance of the biosensor in a range of industrial conditions, we used three experimental scenarios: (i) hybridoma cells grown in shake flasks in DMEM containing phenol red and supplemented with 10% serum and different concentrations of glutamine in the medium to optimize antibody expression level, mimicking the type of medium optimization experiment that is commonly employed in industry; (ii) a CHO‐S cell line expressing a fusion protein grown in batch culture in CD‐CHO medium in shake flasks; and (iii) a GS‐CHO cell line expressing a monoclonal antibody grown in a fed‐batch bioreactor with CD‐CHO medium and fed with CD Efficient™ Feed C AGT™ on alternate days beginning on day 2. Samples were taken over time and simultaneously analyzed using our biosensor workflow, the Bioprofile® Analyzer, and a lactate oxidase enzymatic assay to enable cross validation of the results (Fig. 4a).

Significantly, our results show that the biosensor can accurately determine lactate concentrations in samples from a range of different conditions relevant to the biopharmaceutical industry (Fig. 4b‐d, Table I). Figure 4b shows a snapshot of lactate concentration after 100 h of hybridoma culture in DMEM supplemented with different concentrations of glutamine. Similar types of experiments are done in industry to optimize the formulation of media. Cell number and antibody production vary across the media with different glutamine concentrations (Supplementary Fig. S4) leading to different sample backgrounds, yet the biosensor accurately quantifies the extracellular concentration of lactate when compared with the other assay methods. Similarly, the biosensor provides accurate quantification of lactate accumulation over time in batch culture of CHO‐S in shake flasks (Fig. 4c, Table I) despite changing concentrations of other metabolites (Supplementary Fig. S5). Finally, samples from GS‐CHO cell cultures grown in fed‐batch bioreactor (Fig. 4d, Table I) accumulated much higher concentrations of extracellular lactate over the course of a 16 day culture, which when combined with pH control would lead to very high osmolarity of the samples (Supplementary Fig. S6). Nonetheless, quantification with the biosensor was in good agreement with the other two methods, except for the final sample from 16 days of culture, where the biosensor overestimated the concentration of lactate by a factor of ∼1.5 compared to the average value from the two established methods (Fig. 4d). This overestimate is likely due to the effects of high osmolarity (>700 mmol/L) on cells. Indeed antibody titres have actually decreased on day 16 compared to their maximum at day 12 (Supplementary Fig. S6), suggesting significant amounts of cell lysis have occurred, which would vastly increase the complexity of the sample. Overall, our biosensor has a lower limit of detection for lactate than the BioProfile^®^ Analyzer, which is the current method of choice for metabolite analysis of bioprocessing samples in industry. It is able to measure lactate concentrations as low as 50 μM in M9 medium, which is approximately 100‐fold lower than the lower limit of detection of the BioProfile^®^ Analyzer. Even when samples are in complex backgrounds such as CD‐CHO or DMEM with phenol red, the lower limit of detection was still 100 μM.

## Discussion

We have created a functional whole‐cell lactate biosensor based on the *E. coli* lldPRD operon and have demonstrated that this biosensor can be used for monitoring lactate in complex industrial contexts, specifically mammalian cell cultures producing a recombinant protein, with high accuracy and a lower limit of detection than the industry standard method. In fact, our biosensor was able to detect lactate at a concentration up to two orders of magnitude lower than the Bioprofile analyser (100 μM vs. 1–2 mM) in samples from real cell cultures. To our knowledge this is the first demonstration of robust quantification of samples in complex backgrounds using a whole‐cell biosensor. In addition, our biosensor has a cheaper cost per sample analyzed than the commercial lactate oxidase enzyme assay kit at (approximately 3% of the cost at £13 vs. £403.50 per 96‐well plate analyzed, Supplementary Methods).

The work described here capitalizes on the synthetic biology principles of automation and characterization. We used iterative cycles of characterization of biosensor behavior in increasingly complex conditions to develop a workflow for robust quantification of metabolites in real samples. The workflow we developed can serve as a blueprint for developing whole‐cell biosensors for other metabolites, many of which are important in health and disease (Wishart, 2016), as well as industrial contexts. In this respect, we have shown that biosensors need not be limited to sensing exogenous compounds, which will enable biologists to fully capitalize on the wealth of natural operons available for biosensing to design biosensors for broader variety of compounds. Automation is useful to increase the reproducibility of measurements, permit the detection of small responses, and facilitate the comparison of datasets across different experiments. We also found that culturing the cells in a single base medium (M9 medium) and adding samples of interest gives more robust growth dynamics and better detection ability across many different kinds of samples.

Our whole‐cell lactate biosensor can be applied toward a broad range of biological questions, as lactate is widely used in the food and pharmaceutical industries, and also in the production of polylactic acid, a biodegradable plastic alternative made from renewable resources (Wang et al., 2015). It is also an important clinical biomarker for altered metabolism and many physiological conditions, including inflammation (Haas et al., 2015), cardiovascular diseases (Xie et al., 2016), and cancer (San et al., 2013). Finally, in combination with advances in 3D printing of cells (Cao et al., 2016), we envision that the biosensor could also be used to gain spatial information from adherent cell cultures, which would facilitate the detection of differences in metabolism within individual cells, potentially enabling the diagnosis of diseases such as cancer, artherosclerosis, and diabetes based on metabolic signatures within cells (Galluzzi et al., 2013; Wishart, 2016).

The authors thank Antony Constantinou for providing the CHO‐S cell line for flask samples; Lonza Biologics for the GS‐CHO 46 cell line; Sharmilah Veteryan, Katarzyna Roguska, and Harold Taylor for preliminary work on the whole‐cell lactate biosensor. L.G. was supported by a BBSRC Targeted Priority Studentship. K.M.P. and C.K. were supported by an RCUK Fellowship in Biopharmaceutical Processing. This work was supported, in part, by the EPSRC Frontier Engineering Programme (EP/K038648/1). There are no financial or other conflicts of interest to disclose. The data presented in this paper can be accessed free of charge at: 10.5281/zenodo.266823 and can be used without restriction.

## Nomenclature

CD‐CHO: Chemically defined medium for Chinese Hamster Ovary cell lines
CHO: Chinese Hamster Ovary cells
DMEM: Dulbecco's Modified Eagle Medium
GFP: Green Fluorescent Protein
HPLC: High Performance Liquid Chromatography
MSX: L‐Methionine sulfoximine
PBS: Phosphate buffered saline

## Supporting information

Additional supporting information may be found in the online version of this article at the publisher's web‐site.

**Table S1**. Sequence of LldR promoter
**Table S2**. Primers used for amplification and cloning of LlDR operon components
**Table S3**. Calculation of M9 medium costs

**Figure S1**. Additional lactate biosensor characterization data

**Figure S2**. Time course analysis of biosensor in different mammalian cell culture media.

**Figure S3**. Lactate concentration changes over time in biosensor cultures with different starting lactate concentrations

**Figure S4**. Additional metabolite, cell growth, and antibody production data for hybridoma cell cultures supplemented with different amounts of glutamine

**Figure S5**. Cell growth and metabolite data for the CHO‐S batch flask culture

**Figure S6**. Metabolite and osmolarity data for GS‐CHO fed‐batch bioreactor culture
