# Supplementary material for: Whole‐cell Escherichia coli lactate biosensor for monitoring mammalian cell cultures during biopharmaceutical production
Source: Biotechnol Bioeng. 2017 Feb 23;114(6):1290–300. doi: 10.1002/bit.26254 (PMC5412874; doi:10.1002/bit.26254)
Supplement: Supplementary file 1 — Table S1. Sequence of LldR promoter Table S2. Primers used for amplification and cloning of LlDR operon components Table S3. Calculation of M9 medium costs [file BIT-114-1290-s001.docx]

**Supplementary Tables**

**Supplementary Table 1**: Sequence of LldR promoter

| Promoter | Sequence |
| --- | --- |
| lldPRD promoter | CTTTACCAG ACATCTCCCC CCACAAGAAT TGGCCCTACC AATTCTTCGC TTATCTGACC TCTGGTTCAC AATTTCCCAA TTAAAACTCA CATCAATGTT GCCAATACAT AACATTTAGT TAACCATTCA TTGTCATTAT CCCTACACAA CACAATTGGC AGTGCCACTT TTACACAACG TGTGACAAGG AGATGAGCAA CAGACTCATT ACACGATGTG CGTGGACTCC |

**Supplementary Table 2:** Primers used for amplification and cloning of LlDR operon components

| Primer | Sequence |
| --- | --- |
| LldR Forward | ATGATTGTTTTACCCAGACGC |
| LldR Reverse | TCATGCGTTTTTCTCCCTCG |
| LldR BioBrick Forward | **GTTTCTTCGAATTCGCGGCCGCTTCTAGAG**ATGATTGTTTTACCCAGACGC |
| LldR Biobrick Reverse | **TACTAGTAGCGGCCGCTGCAG**GAAGAAACTCATGCGTTTTTCTCCCTCG |
| LldR Promoter Forward | CTTTACCAGACATCTCCCCCCAC |
| LldR Promoter Reverse | GGAGTCCACGCACATCGTGTAATG |
| LldR Promoter BioBrick Forward | **GTTTCTTCGAATTCGCGGCCGCTTCTAGAG**CTTTACCAGACATCTCCCCCCAC |
| LldR Promoter BioBrick Reverse | **TACTAGTAGCGGCCGCTGCAG**GAAGAAACGGAGTCCACGCACATCGTGTAATG |

Bold indicates BioBrick Prefix and Suffix

**Supplementary Methods: Cost comparison of Biosensor and Lactate Oxidase Assay**

The list price of the lactate oxidase assay kit (Sigma Aldrich, MAK064) was £403.50 on 09/11/2016. The kit will analyse 96 samples (including controls)

For the biosensor assay, for one 96-well plate assay (including controls), the costs included are overnight growth of the biosensor in LB (£1.31) containing 100 μg/mL ampicillin (£0.02), followed by dilution into M9 medium (£0.31) plus ampicillin (£0.04) and IPTG (£0.71), and assay, which uses special tips for the liquid handling system (£10.74) for a total cost of £12.95. The calculation of the M9 costs are shown in Supplementary Table 3. Each assay requires 128 mL of M9 medium for cell growth before sample addition. All chemical prices were taken from the list price of the manufacturer on 09/11/2016 for the product number given in the table.

Both assays require a 96-well plate with clear bottom, the cost of which has been omitted because it is equal in both assays.

**Supplementary Table 3:** Calculation of M9 medium costs

**Supplementary Figure Captions**

**Supplementary Figure 1: Additional lactate biosensor characterisation data** (a) Biosensor response to L-lactate in M9 medium containing glucose as the carbon source. (b) Biosensor response to D-lactate. (c) Biosensor response to pyruvate. Left: Fluorescence normalised by OD600 Middle: Fluorescence rate of change Right: Transfer function from data at 150 minutes. Error bars represent the standard deviation of 6 measurements (2 technical replicates of 3 biological replicates).

**Supplementary Figure 2: Time course analysis of biosensor in different mammalian cell culture media.** (a) Biosensor response to L-lactate when grown in CD-CHO medium. (b-f) Biosensor response to L-lactate spiked into different mammalian cell culture media (b) CD-CHO medium, (c) CD-CHO medium with added HT supplement and 8 mM glutamine, (d) DMEM medium, (e) DMEM medium with added phenol red, (f) DMEM medium with added serum. Left: Fluorescence normalised by OD600 Right: Fluorescence rate of change. Error bars indicate the standard deviation of 6 measurements (2 technical replicates each of 3 biological replicates).

**Supplementary Figure 3: Lactate concentration changes over time in biosensor cultures with different starting lactate concentrations.** Samples were taken at the indicated times after addition of L-lactate and the concentration was measured using the lactate oxidase assay. Error bars represent the standard deviation of 2 technical replicates.

**Supplementary Figure 4: Additional metabolite, cell growth, and antibody production data for hybridoma cell cultures supplemented with different amounts of glutamine.** (a) Viable cell concentration. (b) Percentage cell viability. (c) Integral viable cell concentration. (d) Relative antibody titre after 100 hours of culture normalised to the antibody titre for the culture that was not supplemented with glutamine. (e-h) Extracellular metabolite data (e) Glutamine. (f) Ammonia. (g) Glucose. (h) Lactate. Metabolites were measured using a BioProfile^®^ Analyzer and cells were counted using the trypan blue method and a haemocytometer. Relative mAb concentration was measured using an ELISA. Missing values were above or below the limit of detection of the BioProfile® Analyzer. Error bars for mAb represent the standard deviation of 3 technical replicates.

**Supplementary Figure 5:** **Cell growth and metabolite data for the CHO-S batch flask culture.** Metabolites were measured using a BioProfile^®^ Analyzer and cells were counted using the trypan blue method and a haemocytometer.

**Supplementary Figure 6: Metabolite and osmolarity data for GS-CHO fed-batch bioreactor culture.** Metabolites were measured using a BioProfile^®^ Analyzer and osmolarity was calculated from the following equation: Osm= 1.86([Na^+^] + [K^+^] + [NH4^+^]) + [Glutamate]/ 0.18 + [Lactate]/ 0.096 + constant. Missing values were above or below the limit of detection of the BioProfile^®^ Analyzer.
