# Supplementary figures and images for: Whole‐cell Escherichia coli lactate biosensor for monitoring mammalian cell cultures during biopharmaceutical production
Source: Biotechnol Bioeng. 2017 Feb 23;114(6):1290–300. doi: 10.1002/bit.26254 (PMC5412874; doi:10.1002/bit.26254)

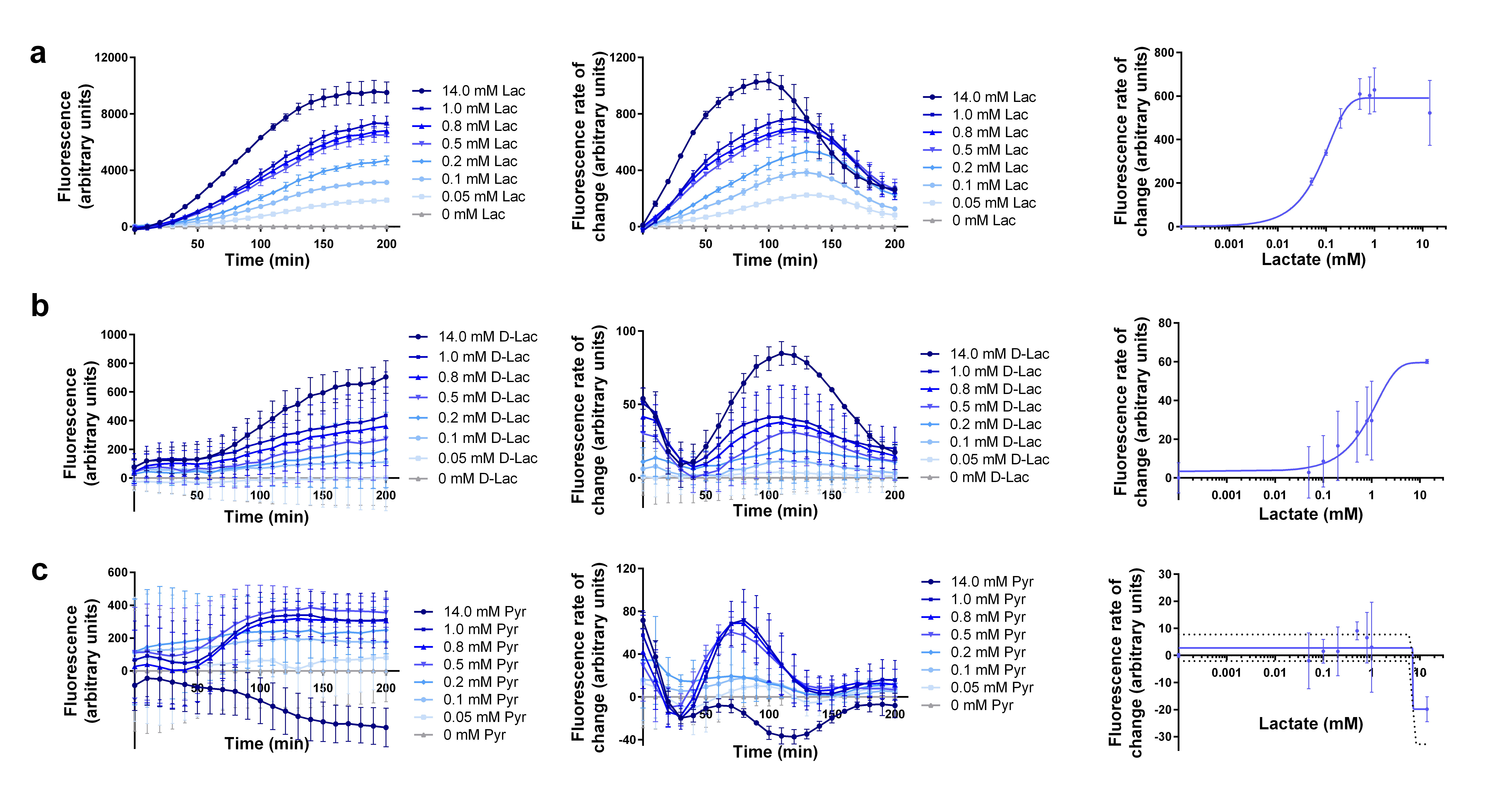

Supplement: Supplementary file 2 — Figure S1. Additional lactate biosensor characterization data [file BIT-114-1290-s002.tif]

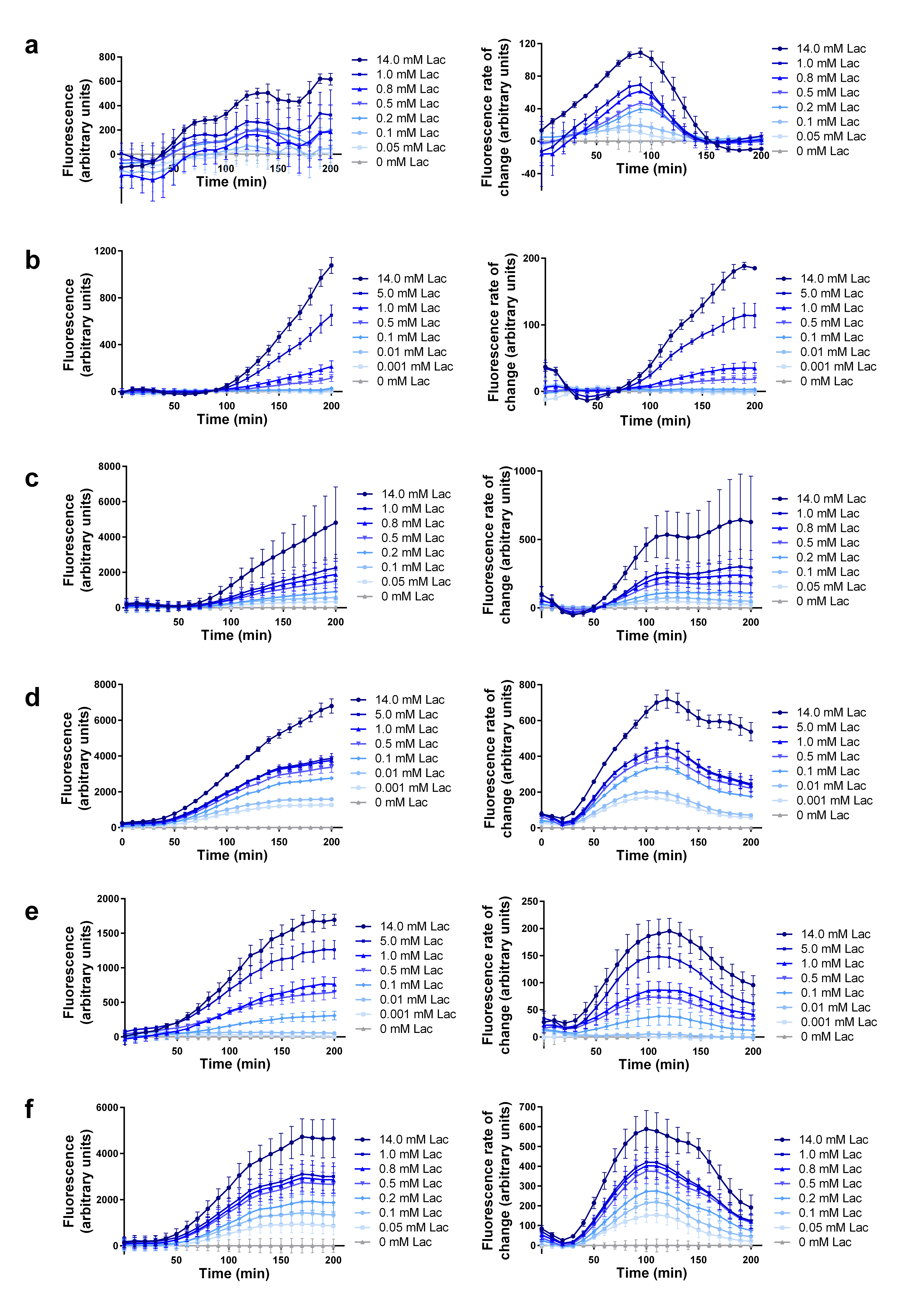

Supplement: Supplementary file 3 — Figure S2. Time course analysis of biosensor in different mammalian cell culture media. [file BIT-114-1290-s003.tif]

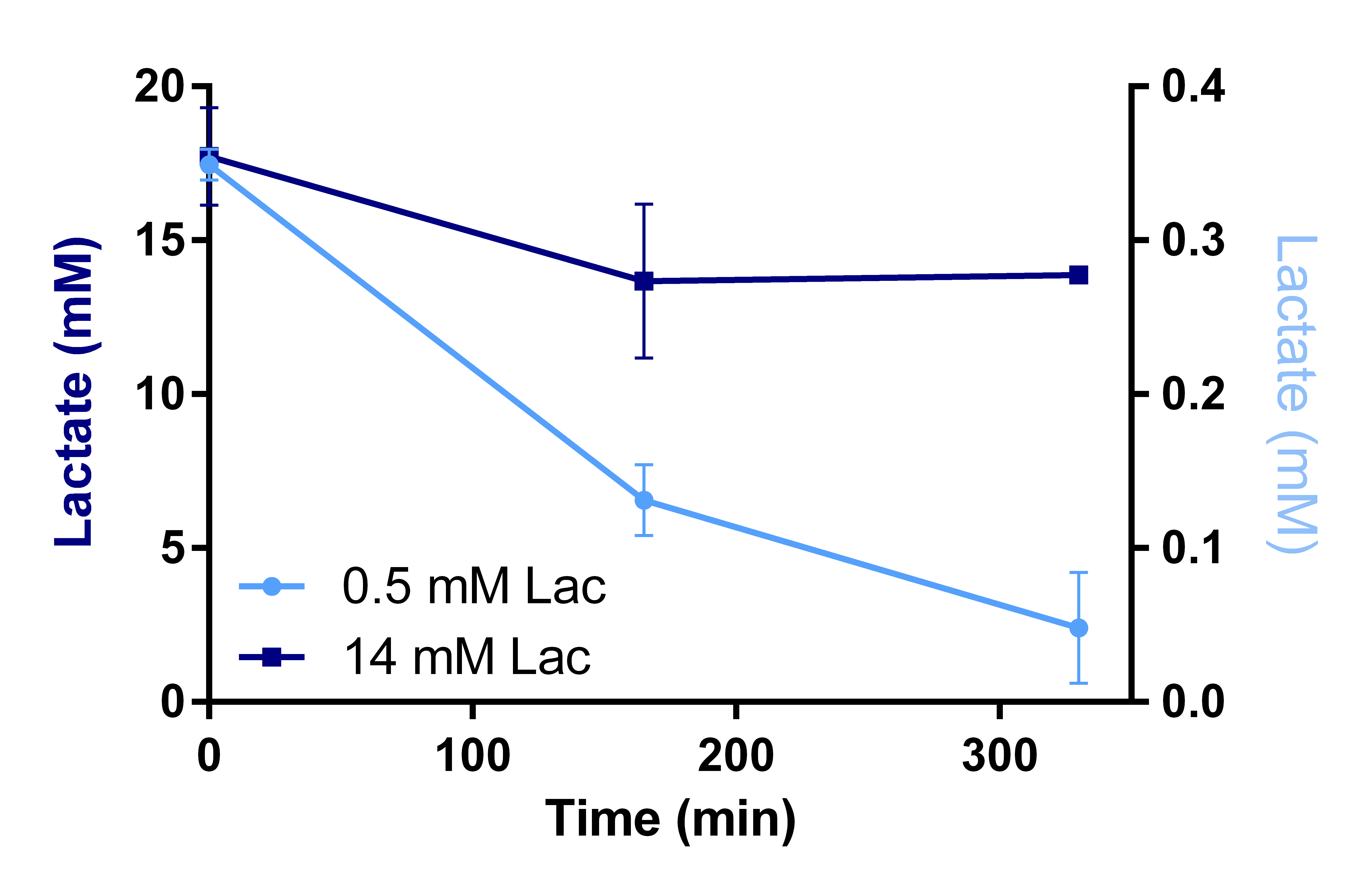

Supplement: Supplementary file 4 — Figure S3. Lactate concentration changes over time in biosensor cultures with different starting lactate concentrations [file BIT-114-1290-s004.tif]

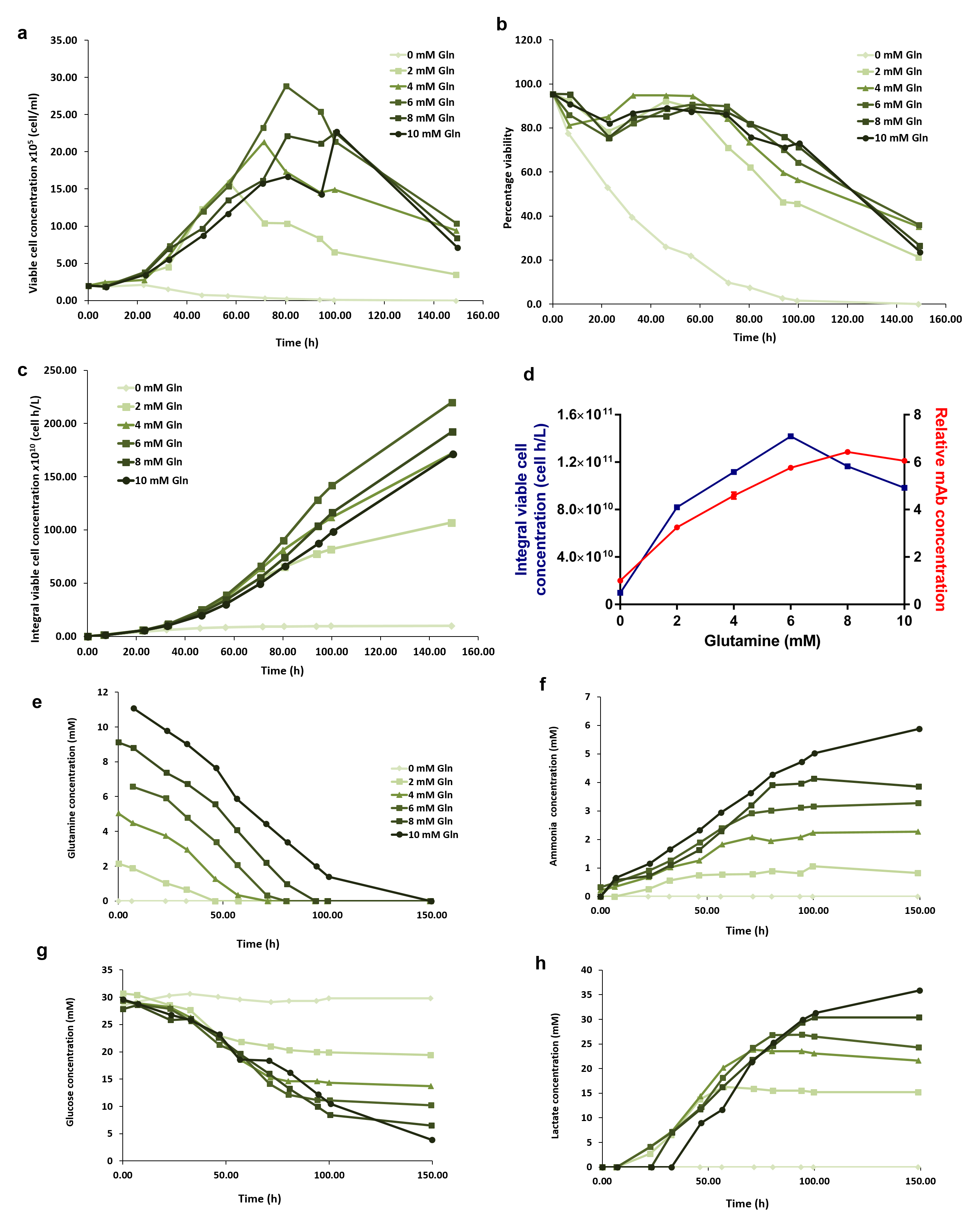

Supplement: Supplementary file 5 — Figure S4. Additional metabolite, cell growth, and antibody production data for hybridoma cell cultures supplemented with different amounts of glutamine [file BIT-114-1290-s005.tif]

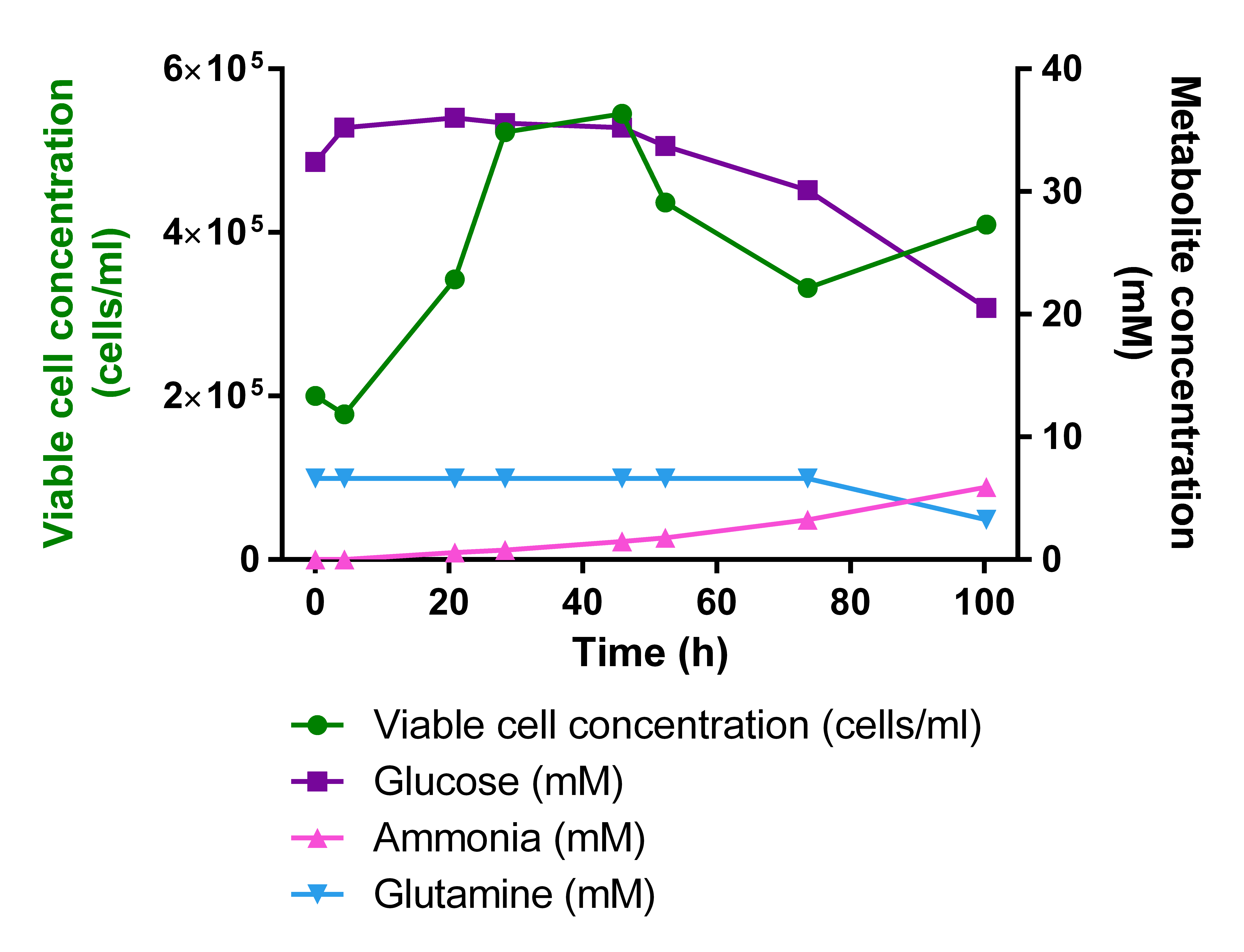

Supplement: Supplementary file 6 — Figure S5. Cell growth and metabolite data for the CHO‐S batch flask culture [file BIT-114-1290-s006.tif]

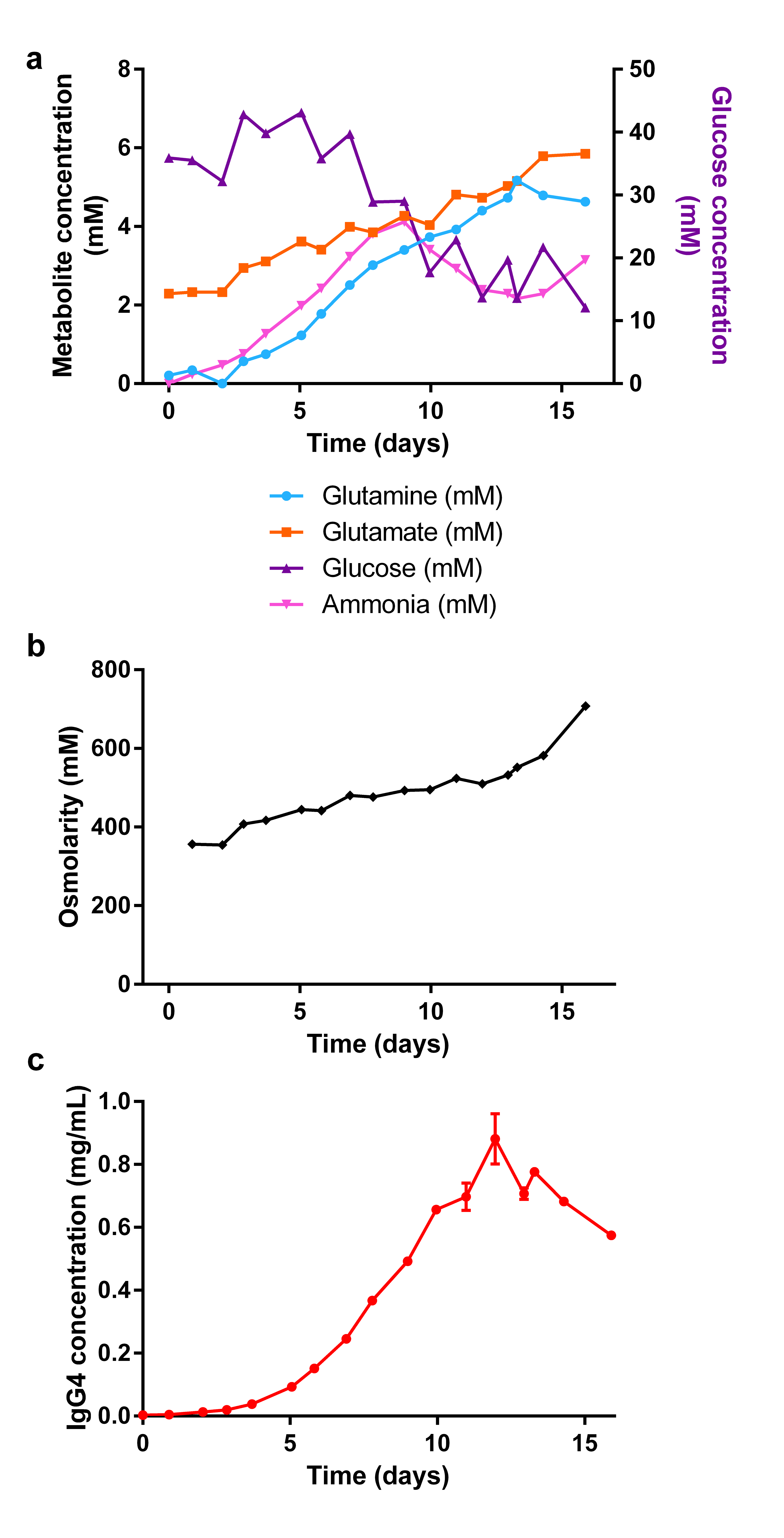

Supplement: Supplementary file 7 — Figure S6. Metabolite and osmolarity data for GS‐CHO fed‐batch bioreactor culture [file BIT-114-1290-s007.tif]
